# The Study and Practice of Homœopathy

**Published:** 1882-06

**Authors:** David Wilson

**Affiliations:** London, England


### A FEW THOUGHTS ON THE STUDY AND PRACTICE OF HOMŒOPATHY.

(*Continued from page 224.*)

David Wilson, M. D., London, England.

We shall now inquire how the examination of the sick requires to be conducted, which, in my opinion, strikes at the root of the whole matter under discussion. Here we have full scope for our anatomical, physiological, histological, pathological, chemical and scientific knowledge generally. Bœnninghausen informs us that before he devoted himself to the study of "that great science," Homœopathy, he had previously prepared himself "by studying with great care the natural sciences and even the old system of medicine." Does not this give us some clue to his great success? Yet even that great man and scholar, thus trained, and who possessed the requisite talent and perseverance for his praiseworthy task, tells us he found it a very difficult business to study some valuable drugs that had not been thoroughly proved in reference to peculiar conditions, and he had to supply the deficiency by contrasting the totality of the symptoms of various drugs, and by studying the genius of a drug from its symptoms. Dr. Carroll Dunham, in the article to which I have referred in the *Philadelphia Journal of Homœopathy*, says of this great man: "Certainly, whether he studied in youth, or in middle age, when his faculties were more mature, I have never conversed with a medical man more learned."

I must be excused for thus dwelling upon the merits of Bœnninghausen, because he also had to fight for the truth amongst the minorities; moreover, in my opinion, his works are the very best that can be put into the hands of the student who desires to study Homœopathy thoroughly, and to practice it in accordance with the strict rules of Hahnemann. By drug provings we have learned the general forms and variety of the individual constitution. For this, adds Grauvogl, "Hahnemann again laid the foundation by his doctrine of *concomitant circumstances*, and no one has understood how to carry out more strictly the consideration of the indication from these circumstances than Dr. von Bœnninghausen. His *Therapeutic Pocket-book* (Münster, Coppenrath, 1846), is an imperishable work of the greatest importance for practice, and could be prepared only by an eminent intellect, and by unwearied theoretical and practical studies." On this I will venture to add a slight correction, as the same *Therapeutic Pocket-book* (Taschenbuch) has been referred to in the introduction to Dr. C. Hering's *Analytical Therapeutics* as Bœnninghausen's *Repertory*. This is an error, and seems strangely inexplicable how it should have escaped Dr. Hering's notice. No doubt the *Taschenbuch*, as its title-page implies, is intended for the sick-room as well as the study of materia medica; but Bœnninghausen told me, most impressively, when I conversed with him in Münster in 1850 that he had compiled it chiefly for the *latter* purpose; whereas his *Repertory*, to which he was then adding copiously from his extensive practice, was really the proper work to be used when prescribing for the sick. He said he should leave his sons to publish a new edition of that work, and it is greatly to be deplored that they have not done so since their father's death. We know that Hahenmann used Bœnninghausen's *Repertory* entirely, and that he considered it indispensable.

The *Taschenbuch* is unquestionably of great value in the sick-room, when used by one who has a fair knowledge of materia medica and who is blessed with a retentive memory. For the examination of the patient, however, I most unhesitatingly declare a good repertory to be indispensable and I do not believe the physician exists who can honestly examine a patient properly without such help. This can be readily illustrated from any rubric we may select. Let us take, for instance, the stomach and other viscera organs of digestion and assimilation and glance over the symptoms as registered in the *British Repertory* under appetite, taste and digestion, or under acidity, eructations, hiccough, nausea and vomiting, each symptom in connection with either of these being composed of sundry elements under diversified rubrics or headings, demanding patient investigation before we can prescribe successfully. We will take, for example, the rubric *Aufstossen*, eructations, with all their concomitants, and let us see whether there be a single practitioner who can retain mentally all these details, and unless he can command them when examining an ordinary case, even of stomach derangement, in which almost all our troubles originate, I defy him to do his duty so as to make an appropriate and strictly homœopathic selection, simple as the epithet *dyspepsia* may seem. Under the rubric *Aufstossen*, then, we have in one of our repertories upwards of 250 subdivisions and these are not all, as Dr. Allen's *Materia Medica* will prove when complete, including time of occurrence, conditions, circumstances and concomitants, etc. And many of these subdivisions include different remedies which must be examined in the materia medica itself, when we have, through these general indications, noted the remedies that correspond in the greatest compass to the individualities of the case before us.

Now let us see what Hahnemann says: "While inquiring into the state of chronic diseases the particular circumstances of the patient with regard to his ordinary occupation, his usual mode of living and diet, his domestic situation and so forth, must be well considered and scrutinized to ascertain what there is in them that may tend to produce or to maintain disease in order that by their removal the recovery may be promoted." (Dr. Dudgeon's translation of *Organon.*) Again, in the *Chronic Diseases*, Hahnemann says, regarding the *proper selection of the remedy:* "The first duty of the homœopathic physician who appreciates the dignity of his character and the value of human life is, to inquire into the whole condition of the patient, the cause of the disease as far as the patient remembers it, his mode of life, the nature of his mind, the tone and character of his sentiments, his physical constitution and especially the symptoms of the disease. This inquiry is made according to the rules laid down in the *Organon.* This being done, the physician then tries to discover the true homœopathic remedy. He may (I say *must*, D. W.) avail himself of the existing repertories with a view of becoming approximately acquainted with the true remedy. But, inasmuch as these repertories only contain general indications, it is necessary that the remedies which the physician finds indicated in those works should be afterwards studied out in the materia medica. A physician who is not willing to take this trouble, but who contents himself with the *general* indications furnished by the repertories and who, by means of these general indications, dispatches one patient after the other, deserves not the name of a true homœopathist."

I repeat that without a repertory we cannot properly interrogate our patients, many of whom are burdened with symptoms of which they only become aware when elicited by the capable examiner. This is often a very difficult undertaking, requiring great tact. The investigation of cases of long standing must be undertaken with great care and conducted as circumstantially as possible, the most *minute* peculiarities must be attended to in chronic diseases, because the most characteristic and cannot be to accurately noted, "partly because the patients become so used to their long sufferings that they give no heed to the lesser accessory symptoms, which are frequently very pregnant of meaning (characteristic), often very useful in determining the choice of the remedy and regard them almost as a necessary part of their condition, almost as health, the real feeling of which they have well nigh forgotten in their sometimes 15 or 20 years of suffering and they can scarcely bring themselves to believe that these accessory symptoms, these greater or lesser deviations from the healthy state, can have any connection with their principal malady." Who has not met with patients who have told us at first that their appetites were excellent, but after a few doses discovered that they were eating much better than formerly? I could give numerous cases of proof of what Hahnemann has so profoundly said, but all medical men must have had ample experience of these facts. Finally, it may be asked whether I really mean to contend that these rules of Hahnemann, on which so much stress is laid, have not been observed by the bulk of homœopathic practitioners? Yes, I do most emphatically declare this to be my honest conviction—a very painful one—and those who question the fact have only to converse with the public, the profession and homœopathic journals, where they will learn more than I have set forth.

In conclusion, I would earnestly recommend that practitioners make themselves thoroughly well acquainted with Bœnninghausen's *Concordances*. I have derived the greatest help from them in practice. While the laborious volumes of Dr. Allen are issuing from the press, I would also recommend each one to construct a repertory for himself on the plan so well executed by Dr. Esrey to the first volume of *Transactions* of the American Institute of Homœopathy. This will prove an invaluable line of study, by which great command will be acquired in the knowledge and use of materia medica. Similar groups of different drugs can be classified under the same rubric, their similarities and differences noted with conditions and concomitant circumstances belonging to each. This is also the line of study I would impress upon the student, very different to that which is now being promulgated in order to enlist converts. Considerable experience in giving chemical instruction to gentlemen who attended my practice at the late Hahnemann Hospital as well as at my own free dispensary during the last 25 years, has satisfied me that very few will devote themselves to hard study, such as the materia medica requires. This is more especially the case if they are already in possession of an allopathic legal qualification. They are contented with a superficial smattering in too many instances. D. C. Hering has pertinently remarked: "Young homœopaths get their stereotyped notions from their earliest acquaintance with the subject and at last cannot conceive of any other way of thinking." This is perfectly true and so many beginners fall into routine habits, when no longer under our observation, that *teachers* cannot be too careful how they initiate students and inquiries in the proper course of study. I have made it a rule for some time to admit no one to clinical study in dispensary practice until he has read the *Organon* of Hahnemann and so far prepared himself to understand the principles of homœopathic action. The late Dr. von Bœnninghausen told me in 1850 that he had read the *Organon* 15 times, and that on each occasion he always acquired something new and valuable. Almost every page is rich in precept and the student is constantly reminded that the only thing is the *totality* of the symptoms; the *sole* thing, in fact, which the physician has to take note of in every case of disease. The whole of the *perceptible* signs and symptoms which we can observe, expressing themselves through *sensations* and *functions*, must be the *sole indication* to guide us in the choice of a curative remedy.

To multiply quotations to the same invariable effect would be useless waste of time, if not an insult to our understanding. Nevertheless, with these plain facts before our eyes all through the *Organon*, the students or audience, at an introductory lecture delivered at the London homœopathic hospital and published in the *British Journal of Homœopathy*, January, 1876, were deliberately told that "a treatment founded on a 'totality of the symptoms' alone, must fail to give us the true indications for treatment. Simple symptom-treatment will fail to enable us to judge between pleurisy and rheumatism of the intercostal muscles, or between pneumonia and bronchitis. It will not give us any indication for treatment in Bright's disease. At the same time ordinary diagnosis alone will not suffice to enable us to treat a case successfully by homœopathic therapeutics, unless we also consider the symptoms presented by each individual case, *i. e.*, we cannot treat disease by specific remedies, according to its name; but, having defined the disease by an accurate diagnosis, we then turn to our reportory, and find the varied morbid states which are likely to be presented to our notice in individual cases, each set forth under the head of the medicine which has power to induce these states, and we select the remedy from one or other of these medicinal drugs, taking that which most closely corresponds to the morbid state of the patient we are about to treat; not according to the name of the disease alone. Our diagnosis reveals the morbid species, but our collection and arrangement of the symptoms enables us to individualize the exact tract, part or organ most needing strength and support, to enable it to recover its last tone, and to rally it from its state of disease." The last sentence clearly informs us that the lecturer considers it necessary to theorize upon the nature of the malady before we have recourse to our repertories, forgetting that Homœopathy is a theory of *cure* (*similia similibus curantur*), and not a theory of *disease*. Besides, the latter proceeding immediately admits into a pure practice an intermediate theory, involving us in the most unsatisfactory speculations, against which Hahnemann very properly and wisely turned his back. The lecturer says: "Perhaps the most complete introductory to the study of Homœopathy is to be found in *Sharp's Essays;* but every one wishing *seriously* to study the system should read Hahnemann's *Organon*, his *Chronic Diseases*, and *Lesser Writings;* he should, however, read them (as he would Hippocrates, Celsus, or Sydenham), as the classics of the system he is about to investigate, and should remember that these works were written before pathology had a right to be called a science." The latter part of this sentence also demands a little serious attention. Surely no one who possessed a proper self-respect and a conscientious sense of his responsibilities when human life might be in his hand would undertake important studies in any other light than *seriously.* Why any apology should have been deemed necessary for Hahnemann's standard works on the pretext that at the period they were written pathology was at a low ebb, does not appear quite satisfactory. It is surely not meant to be implied that, as an advance has been assumed to have taken place in allopathic practice since the days of Hippocrates, Celsus and Sydenham, which many very able allopathic physicians and surgeons of the present day question, therefore the doctrines and practices of Hahnemann have been improved upon by the modern *pseudo*-homœopaths. We have in this paper very cursorily glanced at his *Organon*, by which Homœopathy must stand or fall. His

*Lesser Writings* preceded the *Organon*, and form much of its basis. The first volume of *Chronic Diseases* is a continuation of the principles enunciated in the *Organon*, and the other four volumes contain the symptoms of 49 remedies of priceless value. Although many of these symptoms have been acquired through clinical experience, capable practitioners have confirmed their reliability and worth. Dr. Drysdale's apposite remark recurs to me here very forcibly, for though many other matters may undergo change, "the effects of drugs on the body (as he says) are immutable and unchangeable, and a plain and faithful description of them can never become antiquated or other than correct." It gives me great pleasure to quote Dr. Drysdale when he is dealing with matters of fact; and this seems an opportune moment.

Regarding the provings made and collected by Hahnemann in his *Materia Medica*, now undergoing revision and additions by Dr. T. F. Allen, Dr. Drysdale very significantly writes in the introduction to the Hahnemann *Materia Medica*, Part I, 1852: "How is it that we have got on so well with Hahnemann's earlier provings? To this I reply: 1st, They are good provings, and the symptoms were really produced by the medicines. 2d, A great many of his numbered paragraphs consist of single symptoms and small groups of symptoms, which really occurred in an independent and isolated form from the action of moderately small doses. They are, therefore, short cases of disease reduced to its most essential elementary symptoms, and divested of all merely sympathetic symptoms or after-effects. (See *Organon*, § 137). 3d, Where he (Hahnemann) has split up larger groups, he has for the most part given us the key in the introductory remarks, or the same has been done by the thousand-fold repeated experience of homœopathic practitioners of the *usus in morbus*." The beginner would, indeed, have a feeble mind, not all fitted up for homœopathic study, who might be apt to have it confused by such plain, valuable reading as Hahnemann's *Materia Medica*, which, with the larger editions of the *Symptomatology*, the lecturer informs us, "are all useful as works of reference," but "are apt to confuse the beginner." In their stead *Hull* or Snelling's *Jahr* is recommended, in two volumes, "the one containing the symptomatology, *i. e.*, a record of the symptom induced by each medicine, the medicinal drugs being catalogued in their alphabetical order; the other volume being the repertory, in which the diseases are named, and each drug which is likely to be useful in a disease is pointed out. Few symptoms of disease ever come before us which are not to be found in these volumes, and, what is more, few combinations come before us which are not here set forth." I only wish for the well being of humanity that homœopathic study could be reduced to such a nutshell compass. But my own experience has led me to the opposite conclusion. It is by such works as these being placed in the hands of educated gentlemen that so much ridicule is cast upon Homœopathy; whereas if they were to read Hahnemann's own *Materia Medica* and compare "the short cases of disease reduced to its most elementary symptoms" in the numbered paragraphs, and compare them with similar groups in other drugs all through the *Materia Medica*, they would then take a physiological and pathological interest in such reading, and be enabled to make their own comparisons amongst similarities and differences. They would soon learn to discover the value of conditions and circumstances. They would even begin to see the need of a complete repertory to help them in the examination of the sick, so as to elicit what the patient himself has failed to reveal. The student will learn from the *Organon* the detailed minutiæ that requires to be gone into. (See notes to §§ 87 to 94 of *Organon*.) Those notes also inform the provers how accurately they must observe all their symptoms whilst making trials with various drugs. In § 133 we read, "On experiencing any particular sensation from the medicine, it is indeed necessary, in order to determine the exact character of the symptom, to assume various positions while it lasts, and to observe whether, by moving the part affected, by walking in the room or the open air, by standing, sitting or lying, the symptom is increased, diminished or removed, and whether it returns on again assuming the position in which it was first observed; whether it is altered by eating or drinking, or by any other condition, or by speaking, coughing, sneezing, or any other action of the body; and at the same time to note at what time of the day or night it usually occurs in the most marked manner, whereby what is peculiar to and characteristic of each symptom will become apparent."

To do full justice to this matter I should require to cite the entire *Organon* of Hahnemann. Let an intelligent student read any of Hahnemann's polychrest remedies already published in Dr. Allen's *Materia Medica*, and compare the same with the symptomatology recorded in Hull or Snelling's *Jahr*, and he will soon discover how the remedies have been pruned in the latter works of most important, characteristics, and each drug "presented to us a short, compact, stemless stick, all ready to be tied in a faggot." In this cursory view of matters I must not omit to take notice of Pathology—a very big word amongst us—but I should like to know what service has been rendered to Homœopathy by all the progress it may have made since the time of Hahnemann? None, whatever, in my opinion. Moreover, it would be well that those who make frequent use of the word should state what they mean, seeing that it has a double signification. A very learned allopathic writer says: "When I speak of the pathology of a disease I do not mean those obvious alterations in the structure of an organ which we meet with in the *post-mortem* examinations, but the so-called functional changes which precede and are the cause of both them and the symptoms." Will any one who has read the *Organon* attentively, make so bold as to deny that Hahnemann did not attend to these "functional changes" which reveal themselves by signs and symptoms indicating the remedy needed, if we know *how* and *where* to search for it? It was in this latter sense, I presume, that Dr. C. Hering wrote: "Not only is a study of pathology indispensable, but its employment also, in the investigation of every actual case of disease, both for diagnosis, prognosis and prophylaxis. We differ essentially, however, if, on the one hand, we conduct the examination of the patient on the true Hahnemannian plan, and choose the best remedy according to the law of the similarity of the symptoms; or if, on the other, we make only a pathological examination, determine the nature of the disease and then administer the remedy that is set opposite that name in the repertoires." He further adds: "It will be found that all those who use high potencies examine the patient in the Hahnemannian fashion and choose the remedy from the symptomatic indication; while those, on the other hand, who are entirely or mainly led by the pathological indications, rely wholly or principally upon the lower attenuation." If the lecturer had in his mind the pathology implying "alterations in the structure," such as have too often taken place in diabetes and Bright's disease before the sufferers seek homœopathic treatment, then we would beg to remind him that Homœopathy does not profess any more than Allopathy to cure organs that have already passed into states of disorganization, although, in this respect even, I have found remedies selected according to the symptoms afford the greatest relief. When not guided by the symptomatic indications all becomes guess-work and chaos.

I have made my strictures with less reserve than I might otherwise have done had I not been aware, through the *British Journal of Homœopathy*, that amongst the approved papers contributed to the World's Homœopathic Convention by five members of the British Homœopathic Society, *two* of them prepared by lecturers at the hospital, have reference, respectively, to "Homœopathic Education," and "Homœopathic Literature," subjects of vital importance to Homœopathy, demanding not only earnest thought but energetic action. The hospital and society are united as one body, under a rule which I have formerly discussed in my *History of the London Homœopathic Hospital.* I trust it will be found that my opinions are based on facts, without any wavering or dubious uncertainty, differing entirely, in my judgment, from *opinions* which have gained the assent of the understanding by the evidence of *probability* alone. Finally, as a British homœopathic physician, I make this public protest against the unjustifiable falsification of Hahnemann's doctrine and principles of practice, whether adopted in Great Britain, America or elsewhere, until he, our great master, shall have been tested, according to his own rules, and found wanting!

